# Guidelines, Counseling Practices, and Fertility Preservation Options for Childhood Cancer Patients in the Nordic-Baltic Region

**DOI:** 10.1200/OP-24-00623

**Published:** 2025-04-04

**Authors:** Monika Grubliauskaite, Zivile Gudleviciene, Irma C. Oskam, Babak Asadi-Azarbaijani, Kirsi Jahnukainen

**Affiliations:** ^1^ Life Sciences Center, Vilnius University, Vilnius, Lithuania; ^2^ Department of Biobank, National Cancer Institute, Vilnius, Lithuania; ^3^ Faculty of Medicine, Vilnius University, Vilnius, Lithuania; ^4^ The Livestock Production Research Centre, Norwegian University of Life Sciences, Ås, Norway; ^5^ Faculty of Health Sciences, VID Specialized University, Oslo, Norway; ^6^ Department of Pediatrics, University of Helsinki and Helsinki University Hospital, Helsinki, Finland; ^7^ NORDFERTIL Research Lab Stockholm, Childhood Cancer Research Unit, Karolinska Institute and Karolinska University Hospital, Stockholm, Sweden

## Abstract

**PURPOSE:**

Despite new medicine and treatment options, fertility is impaired for many childhood cancer survivors after gonadotoxic treatment. In the current study, we compiled an overview on the state of fertility preservation (FP) care and limitations for childhood cancer patients throughout the Nordic-Baltic region.

**MATERIALS AND METHODS:**

In partnership with the Nordic Society of Pediatric Hematology and Oncology, an anonymous survey was conducted among 23 major pediatric oncology centers in Nordic and Baltic countries. The survey featured 22 multiple-choice and open-ended questions that provided insights into guidelines, available FP options, clinical indications, and counseling.

**RESULTS:**

The response rate to the questionnaire was 74% (17 of 23 pediatric oncology centers). The survey revealed that only 65% of the centers have national guidelines on FP at the time. Although all centers offer counseling before treatment by oncologists (88%) or gynecologists (65%), 76% of the centers provide it only to those fulfill inclusion criteria. Additionally, counseling is unavailable for some patients because of age (35%), communication issues (29%), or lack of time (24%). Predominantly, sperm cryopreservation is offered across all centers for pubertal boys, while testicular tissue cryopreservation is provided at 41% of pediatric oncology centers for prepubertal boys. Oocyte cryopreservation is offered to pubertal girls at 88% of the centers, and ovarian tissue cryopreservation is offered to prepubertal and pubertal girls at 82% of the questioned centers.

**CONCLUSION:**

The survey highlights the implementation of FP services status in the Nordic and Baltic countries. However, standardizing FP indications and disseminating guidelines widely is crucial to reduce clinical variability. Addressing issues such as inconsistent counseling, limited collaboration, and unclear risk stratification can drive further improvements.

## INTRODUCTION

As childhood cancer survival rates improve,^1,2^ preserving fertility function post-treatment becomes crucial.^3^ Gonadal damage, often resulting from surgical treatment, radiation, or chemotherapy, profoundly affects survivors' physical and psychosocial quality of life regardless of cancer type.^4^ Fertility counseling and pursuing fertility preservation (FP) have been associated with beneficial psychosocial outcomes, including enhanced quality of life, increased overall satisfaction, and reduced feelings of regret and loneliness.^5-7^

**CONTEXT**

**Key Objective**
To provide an overview of guidelines, counseling practices, and fertility preservation (FP) options for childhood cancer patients in the Nordic-Baltic region.
**Knowledge Generated**
The survey revealed significant variability between centers in patient selection for FP and counseling, on the basis of patient age and maturity, planned treatment exposures, and type of FP method.
**Relevance**
Key elements necessary for improving oncofertility care include guidelines for harmonized clinical practices, as well as specialized training for health care professionals to enhance their expertise and confidence in conducting time-sensitive consultations.

Despite these considerations, a significant number of patients and their families do not receive information about fertility and its preservation options at the time of diagnosis.^3,8^ Studies indicate that health care professionals encounter challenges when discussing fertility-related topics with patients and their guardians,^5,9^ often viewing infertility as a less immediate concern compared with the cancer diagnosis itself.^9^ Although national and international guidelines recommend that health care providers proactively discuss fertility concerns with all patients with cancer and their parents, access to counseling and preservation options varies widely across countries.^10^

The Nordic Society of Pediatric Hematology and Oncology (NOPHO) is a collaborative network of pediatric hematologists, oncologists, and researchers.^11^ Established in 1980 by the Nordic countries, NOPHO expanded in 2016 to include Lithuania, and later Latvia and Estonia, thereby creating a network encompassing the Nordic-Baltic regions dedicated to improving pediatric treatment options and quality of life, including FP.

To our knowledge, for the first time in this study, we examine the current landscape of oncofertility care for children across the Nordic and Baltic countries. The aim of this survey was to promote effective services, harmonized clinical practices, and standardized guidelines throughout the Nordic-Baltic region.

## MATERIALS AND METHODS

In collaboration with NOPHO, an anonymous questionnaire was electronically distributed to the directors of 23 main pediatric oncology centers in the Nordic and Baltic countries. The questionnaires were developed by authors in collaboration with pediatric oncologists and gynecologists to evaluate the status of FP practices for pediatric cancer patients in these regions. The questionnaire consists of 22 multiple-choice and open-ended questions, covering topics such as guidelines, available FP options, clinical indications, the implementation of FP in practice, and counseling (Data Supplement, Table S1, online only). All study centers are public university hospitals with pediatric cancer units. FP services for patients with cancer at all the Nordic and Baltic oncology centers are primarily publicly financed, with support in some cases from facility programs or patient self-payment.

The questionnaires were completed by pediatric oncologists in collaboration with other specialists at pediatric oncology centers. The directors of each center either provided the answers themselves or designated someone within the center to complete the questionnaire. Data were collected anonymously and electronically in 2022 through the web-based online questionnaire “Nettskjema,” which has a data management agreement with the University of Oslo.^12^ Up to three reminders were sent. The results were analyzed using descriptive percentage statistics, with all calculations performed using Excel Microsoft 365.

## RESULTS

A total of 17 pediatric oncology centers responded to the questionnaire, yielding a response rate of 74%. The average clinical experiences of the oncologists who responded was 17 years (3-32 years). A breakdown of the responding centers on national guidelines on FP for patients with cancer, by country, is presented in Table 1. Table 2 shows a perspective view of the main results of this study. At the time of the survey, only the Nordic Network recommendations^13,14^ were available online and in English language.

**TABLE 1.** The Number of Responding Centers to the Survey and the Question If There Are Any National Guidelines on FP for Patients With Cancer in Their Country

Responded to the Survey		Responded to the Question Regarding National Guidelines on FP			
Country	Year 2022	Yes	Do Not Know	No	Total
Denmark	2/4	2	—	—	2
Estonia	1/1	—	—	1	1
Finland	3/5	2	—	1	3
Iceland	1/1	—	—	1	1
Latvia	1/1	—	—	1	1
Lithuania	2/2	2	—	—	2
Norway	3/4	2	1	—	3
Sweden	4/5	3	1	—	4
Total	17/23	11	2	4	17

Abbreviation: FP, fertility preservation.

**TABLE 2.** A Perspective View of the Main Results

Question	Number of Centers/Total (%)
Legislation/guideline	
Choose the male FP options offered in your country	
Sperm cryopreservation	17/17 (100)
Sperm donation	13/17 (76)
Gonadal shielding	11/17 (65)
TTC before puberty	8/17 (47)
Adaptation	6/17 (35)
TESA/PESA	5/17 (29)
TTC after puberty	3/17 (18)
Choose the female FP options offered in your country	
Oocyte cryopreservation	15/17 (88)
OTC before puberty	14/17 (82)
OTC after puberty	14/17 (82)
Gonadal shielding/transposition	12/17 (71)
Egg donation	9/17 (53)
GnRH protocol	7/17 (41)
Adaptation	6/17 (35)
Embryo cryopreservation	5/17 (29)
In vitro maturation	4/17 (24)
Embryo donation	2/17 (12)
Surrogate	0/17 (0)
Does your center have guidelines available regarding	
Sperm collection and preservation?	11/17 (65)
Ovarian cortical tissue collection and preservation?	11/17 (65)
Testicular tissue collection and preservation?	10/17 (59)
Ovarian stimulation and oocyte cryopreservation?	9/17 (53)
We do not have any guidelines	5/17 (29)
It will be provided	0/17 (0)
Choose the FP options offered in your center	
Sperm collection and preservation	14/17 (82)
Ovarian cortical tissue collection and preservation	11/17 (65)
Ovarian stimulation and oocyte cryopreservation	10/17 (59)
Testicular tissue collection and preservation	7/17 (41)
FPs take place in another center	4/17 (24)
We do not offer any of FP options	0/17 (0)
Practice	
Does your center have an established collaboration with service for	
Sperm collection and preservation?	14/17 (82)
Ovarian cortical tissue collection and preservation?	12/17 (71)
Testicular tissue collection and preservation?	11/17 (65)
Ovarian stimulation and oocyte cryopreservation?	10/17 (59)
We do not collaborate with any services	0/17 (0)
It will be provided	0/17 (0)
Do you ever offer sperm/testicular tissue/ovarian cortical tissue/oocyte preservation to individuals with the following diagnosis?	
Hodgkin disease	15/17 (88)
Before stem-cell transplantation	15/17 (88)
Non-Hodgkin lymphoma	14/17 (82)
Ewing's/soft tissue sarcoma	14/17 (82)
Osteosarcoma	14/17 (82)
Acute lymphoblastic leukemia at diagnosis	8/17 (47)
Acute myeloid leukemia at diagnosis	8/17 (47)
Wilms tumors	6/17 (35)
Germ cell tumors	6/17 (35)
CNS tumors	5/17 (29)
Others	2/17 (12)
Do you offer counseling on FP options for patients and parents?	
Yes, only those who fulfill indications for FP	13/17 (76)
Yes, all	2/17 (12)
Yes, some	2/17 (12)
No	0/17 (0)
I do not know	0/17 (0)
Is there any available information about FP for patients and parents before and after cancer treatment?	
Yes	7/17 (41)
It will be provided	5/17 (29)
No	4/17 (24)
Do not know	1/17 (6)
Should be discussed	0/17 (0)
If yes, how do you give information?	
Brochures	7/17 (41)
Online	4/17 (24)
Others	1/17 (6)
Book	0/17 (0)
Combination of all	0/17 (0)
Does your center offer counseling on fertility issues for patients and parents?	
Yes, all patients and parents	8/17 (47)
Yes, some	7/17 (41)
No	2/17 (12)
Do not know	0/17 (0)
Why have not all patients and parents been offered fertility consultation?	
We do not offer fertility consultation to all patients and parents	6/17 (35)
Age (too young/old)	6/17 (35)
Language/communications issues	5/17 (29)
Lack of time for any consultation	4/17 (24)
Other issues are important	3/17 (18)
Patients/parents have not been interested or embarrassed	2/17 (12)
Religion/culture	0/17 (0)
Sexual orientation	0/17 (0)
No information available	0/17 (0)
Who is responsible for fertility consultation?	
Oncologist	15/17 (88)
Gynecologist	11/17 (65)
Endocrinologist	3/17 (18)
Oncology nurse	2/17 (12)
Others	2/17 (12)
Andrologist	1/17 (6)
There is no consultation	0/17 (0)
Male FP	
Who is asking for sperm collection?	
Oncologist	16/17 (94)
Oncology nurse	2/17 (12)
Endocrinologist	2/17 (12)
Andrologist	1/17 (6)
Gynecologist	0/17 (0)
Others	0/17 (0)
How do you decide that a boy is old enough to produce a sperm sample?	
Puberty stage	17/17 (100)
Where is the sperm sample produced?	
At the sperm laboratory	17/17 (100)
At the patient's home	7/17 (41)
In the patient's hospital room	1/17 (6)
Others	1/17 (6)
What do you do if a boy is unable to produce a sperm sample?	
No options	8/17 (47)
Testicular tissue preservation	3/17 (18)
PESA/TESA	3/17 (18)
Electroejaculation	3/17 (18)
Try again ejaculation	2/17 (12)
Do you ever offer sperm/testicular tissue preservation after the cancer treatment?	
No	10/17 (59)
Yes, some patients	7/17 (41)
Yes, for all	0/17 (0)
Do not know	0/17 (0)
Female FP	
Do you ever offer ovarian cortical tissue/oocyte preservation after the cancer treatment?	
Yes, some patients	8/17 (47)
No	8/17 (47)
Yes, for all	1/17 (6)
Do not know	0/17 (0)
Do you have any routines for treating girls who have recovered from ovarian failure?	
No routine	11/17 (65)
Consultation with gynecologist	6/17 (35)
Consultation with endocrinologist	2/17 (12)
Others	0/17 (0)

Abbreviations: FP, fertility preservation; GnRH, gonadotropin-releasing hormone; OTC, ovarian tissue cryopreservation; PESA, percutaneous epididymal sperm aspiration; TESA, testicular sperm aspiration; TTC, testicular tissue cryopreservation.

### Guidelines for FP and Counseling

More than half of the respondents (65%) indicated that their country has established national guidelines on FP for patients with cancer (Table 1). Moreover, all centers (100%) reported having established collaborations with service providers for one or more FP options (Table 2). Upon closer examination, discrepancies were observed regarding the awareness and availability of national guidelines (Table 1). Although some centers reported the existence of approved national guidelines, others within the same country were either unaware of them (eg, Norway and Sweden) or indicated their absence (eg, Finland). Additionally, three countries—Estonia, Iceland, and Latvia—reported the complete absence of approved national FP guidelines.

Altogether, 65% of centers reported having guidelines for one or more FP options on the basis of therapeutic agents, patient age, or other criteria, and there was clear consensus regarding guidelines for offering sperm and oocyte cryopreservation on the basis of pubertal maturation (Data Supplement, Tables S2 and S4). One center indicated that testicular tissue cryopreservation (TTC) is primarily offered for prepubertal boys, while another center reported that ovarian tissue cryopreservation (OTC) for girls younger than 16 years is conducted within a research context (Data Supplement, Table S2). Moreover, only 47% of centers reported having established clinical protocols for the management of girls who have recovered from ovarian failure.

Although responses varied, the majority of the centers indicated that FP would be performed before initiating high gonadotoxic treatments, such as hematopoietic stem-cell transplantation (SCT), irradiation exposing gonads, and in three centers, high-dose alkylating agents (Data Supplement, Table S2). The centers reported variability in the diagnoses for which gamete or reproductive tissue preservation is offered (Data Supplement, Table S3). The majority of centers indicated providing FP options for patients diagnosed with Hodgkin disease (88%), non-Hodgkin lymphoma (82%), Ewing's sarcoma/soft tissue sarcoma (82%), osteosarcoma (82%), and those undergoing SCT (88%; Table 2). By contrast, fewer than half of the centers offered FP for patients with acute lymphoblastic leukemia (45%), acute myeloid leukemia (45%), Wilms tumor (35%), and germ cell tumors (35%). Additionally, two centers specifically mentioned that patients with hematologic malignancies are either not offered the service or require special consideration.

Although the majority (88%) of the centers offered counseling on fertility issues, it remains unavailable for some patients and parents (Table 2). Respondents cited various reasons for this, including the patients' age (35%), language/communications issues (29%), lack of time for consultation (24%), or patients or parents not being interested or feeling embarrassed (12%). All centers reported offering pretreatment counseling on FP options to patients and their parents (Table 2). However, approximately 76% indicated that this counseling is provided only to those meeting the local eligibility criteria for FP. Additionally, only 41% of respondents reported having informational resources available for patients and parents, such as brochures (41%) or online information (24%; Table 2). In the majority cases, counseling was provided by pediatric oncologists (88%) or gynecologists (65%). The remaining centers referred patients and parents to specialists or oncology nurses (Table 2).

### Male FP Practices

TTC was offered to prepubertal males in 41% of the pediatric oncology centers, both before and after cancer treatment. For pubertal boys, all centers (100%) consistently offered sperm banking. Sperm cryopreservation for FP primarily focused on their sexual development. The determination of sexual maturity was based on consultation with patients and parents, clinical examination according to Tanner staging, and assessment of testicular development (Data Supplement, Table S4).

In most centers (94%), pediatric oncologists were primarily responsible for requesting sperm sample collection. However, in some centers, endocrinologists (12%), oncology nurses (12%), or andrologists (6%) could also make this request. Although all respondents reported that sperm samples could be obtained at the sperm laboratory, some centers also allowed collection at the patient's home (6%), in a designated room at the hospital (47%), or other places (6%).

Only nine of the oncology centers (53%) reported alternative methods for sperm collection in cases when mature boys were unable to produce ejaculated samples. These techniques included electroejaculation, testicular sperm aspiration, percutaneous epididymal sperm aspiration, and TTC (Table 2).

### Female FP Practices

The majority of Nordic and Baltic centers offered oocyte cryopreservation (88%), OTC (82%), and gonadal shielding/transposition (71%) as primary FP options for pubertal girls (Table 2). Hormonal treatment to potentially protect the ovaries from chemotherapy was used only in 41% of oncology centers. Additionally, OTC for prepubertal girls was provided by 82% of pediatric oncology centers.

Oocyte cryopreservation and OTC have also been offered after cancer treatment by 53% of oncology centers.

## DISCUSSION

This study provides a comprehensive overview of guideline awareness, counseling practices, and FP options and key limitations in oncofertility care across the Nordic and Baltic countries. The survey demonstrated active implementation of FP services for children in all surveyed regions. Oocyte cryopreservation and OTC were offered by over 80% of centers, while sperm cryopreservation was available in all centers and TTC in 40% of the centers. Notably, significant variability was observed among centers regarding eligibility criteria for FP services and awareness of national guidelines. Although the majority of centers acknowledged the existence of national guidelines, some reported being unaware of them, and others indicated their absence. This finding indicates a significant gap in the dissemination and awareness of national guidelines, underscoring the need for enhanced communication and systematic implementation of FP protocols across pediatric oncology centers in the Nordic and Baltic regions.

Most of the surveyed countries are following Nordic Network recommendations for FP of prepubertal and pubertal patients.^13,14^ However, recently, a study has been published indicating that in Lithuania, at least one of the pediatric oncology centers started implementing recommendations from PanCareLIFE consortium and The International Late Effects of Childhood Cancer Guideline Harmonization Group.^15^ No information of changes in FP in Latvia or Estonia has been reported. Nordic Network guidelines provide overarching recommendations for FP eligibility for pediatric age groups. According to the guidelines, prepubertal children who are facing oncologic treatments associated with a very high risk of infertility could be offered the experimental procedure of gonadal tissue cryopreservation, while adaptation to adult indications is encouraged for pubertal and postpubertal children. The guidelines specify that treatments associated with a very high risk of infertility include allogeneic/autologous SCT or radiotherapy involving the gonadal region. There is clear international consensus that myeloablative conditioning for bone marrow transplantation and direct gonadal radiation carry a significant risk of infertility, and FP should be considered.^16,17^ The present survey confirmed that these therapies were commonly agreed as indication for FP in the Nordic and Baltic countries also.

Compared with other international FP guidelines, the Nordic Network guidelines do not specify eligibility for FP on the basis of exposure to alkylating agents. European PanCareLIFE guidelines give moderate recommendations of OTC to prepubertal girls exposed to >6,000-8,000 mg/m^2^ cumulative cyclophosphamide equivalent dose (CED),^16^ and prepubertal boys exposed to >4,000 mg/m^2^ CED.^17^ Similarly, the American Oncofertility Consortium identifies exposure to >4,000 mg/m^2^ CED for boys, >12,000 mg/m^2^ for prepubertal girls, and >8,000 mg/m^2^ for pubertal girls associated with a high risk of future gonadal insufficiency or infertility.^18^ In the present survey, one center reported offering OTC for girls receiving alkylating agent therapy >6 g/m^2^ or sperm cryopreservation for boys receiving high-dose alkylating agents >4 g/m^2^, potentially reflecting these international risk stratification thresholds. The ambiguity in the risk stratification of alkylating agents likely underlies the observed variability in the interpretation of FP eligibility across Nordic and Baltic countries. For example, the Swedish national recommendations include specific threshold doses for chemotherapies associated with a high risk of infertility and state that pubertal and postpubertal children receiving these therapies are eligible for FP.^13,14^ By contrast, the Finnish national recommendations do not include such threshold doses.^19^ This observation emphasizes the importance of establishing a standardized consensus on FP indications and a robust risk stratification system to achieve harmonized clinical practice. Challenges in developing such risk stratification model for children include the lack of robust clinical evidence defining precise threshold doses for sterilizing childhood chemotherapies,^20^ as well as the limitations of the CED scoring framework, which excludes several alkylating chemotherapeutic agents commonly used in pediatric oncology, including dacarbazine, temozolomide, and treosulfan.^21^

Although there is clear consensus that counseling is an essential component of FP, the present survey revealed considerable ambiguity regarding who were counseled, who provide the counseling, and how the specific information was delivered. Two centers provided fertility counseling to all pediatric patients, whereas others limit this service to those undergoing high-risk therapies associated with subfertility. Several reasons were reported for why this information was not provided, including the patient's age, language or communication barriers, time constraints, and a lack of interest or feelings of embarrassment from patients or parents. Health care providers must carefully consider the nature of the treatment, the overall health status of the patient, and the potential risks and benefits of FP, especially when dealing with minors, to optimize patient selection for FP. The survey highlights that ambiguity regarding FP indications may also contribute to reduced counseling activity.

Our study found that oncologists or gynecologists usually handle fertility consultations in the Nordic and Baltic countries. The Nordic network recommendations suggest that if FP could be offered, the information should be provided by a professional, specifically trained for this purpose.^13,14^ Patients appreciate if the counseling on FP is initiated by the health care providers^22,23^; however, it is reported that the lack of knowledge on FP and selection of patients makes them feel discomfort and unwillingness to discuss it with young patients with cancer.^9,24^ In prepubertal patients, the experimental nature of FP, as noted in the responses of the present survey, may contribute to health care professionals' reluctance to initiate discussions on this topic. Providing written information about FP, such as patient brochures or pamphlets, can be highly beneficial, as patients and parents may feel overwhelmed by the volume of information presented immediately after diagnosis. This is supported by findings from seven previous studies (total of 146 patients) where 45% of patients did not recall receiving any information on fertility.^23^ Similarly, only 41% of Nordic and Baltic pediatric oncology centers reported having informational resources, such as brochures or online materials, available for patients and parents.

Another identified gap in clinical practice was the lack of systematic collaboration between oncology and reproductive centers in the Nordic and Baltic countries. The surveyed centers primarily used FP services provided by nearby university hospitals. However, these services may have been limited to specific FP programs or single FP methods. The lack of centralization may also limit patients' access to the full range of available techniques. Enhanced collaboration among Nordic and Baltic countries could facilitate joint research initiatives, improve resource sharing, and expand specialist expertise, as demonstrated by programs such as the Nordic NORDFERTIL FP initiative for prepubertal boys^25^ and the German FertiPROTEKT network for young females.^26^

The present survey demonstrates that standard-of-care FP options, such as sperm and oocyte cryopreservation, are well established for postpubertal patients in the Nordic and Baltic countries. However, considerable variability in FP practices remains, with notable differences observed in the use of gonadotropin-releasing hormone antagonists (GnRHa) for female patients. No association has been found between the use of GnRHa during chemotherapy and higher rates of childbirth, natural conception, maintained fertility, or reduced cancer mortality.^27^ This aligns with the lack of recommendations from ESHRE,^28^ PanCareLIFE,^16^ and the Nordic Network.^13^ Despite this, seven pediatric oncology centers reported offering this FP option. Our survey found that only half of the centers have established routines for treating postpubertal girls who have recovered from ovarian failure. This indicates that follow-up clinics to timely identify ovarian recovery are established in only half of the Nordic and Baltic centers. Although all surveyed oncology centers offer sperm cryopreservation for pubertal boys, factors such as primary disease,^29^ anxiety, or religious and cultural considerations may prevent some patients from providing sperm samples.^30^ As alternatives, electroejaculation, surgical sperm extraction with possible sperm collection, or TTC can be considered for high-risk patients, according to PanCareLIFE^17^ and Nordic Network guidelines.^14^ Only 53% of centers reported the availability of these alternative methods in their unit for pubertal boys.

A major drawback of current FP options using cryopreserved gonadal tissue is the risk of reintroducing malignant cells during autologous transplantation.^31^ Most solid tumors may have a lower risk of metastasis to reproductive tissues compared with hematologic malignancies. In our survey, we discovered that nearly 50% of centers reported cryopreserving gonadal tissue for patients with acute leukemia. For these children, fertility options using cryopreserved tissue may be limited because of the lack of a reliable clinical technique to exclude malignant contamination. Consistent with this, two centers reported that patients with hematologic malignancies are either not offered the service or require special consideration. Comprehensive counseling is crucial to inform all prepubertal patients and families about the experimental nature and associated uncertainties of gonadal tissue cryopreservation, particularly in cases with a risk of cancer cell contamination in the gonadal tissue.

In conclusion, this analysis highlights the active implementation of FP services across the Nordic and Baltic countries. It also identifies key elements necessary for successful oncofertility care. Establishing a standardized consensus on FP indications is critical to supporting health care providers in delivering effective FP services. Broad dissemination of these guidelines is necessary to reduce variability in clinical practice. Further progress can be achieved by addressing factors contributing to the observed heterogeneity in FP practices, such as inconsistencies in counseling protocols, limited collaboration between oncology and reproductive centers restricting FP options, and ambiguity in risk stratification. Ultimately, increased international collaborative research is needed to address knowledge gaps, particularly regarding risk stratification for alkylating agents.
